# Pleiotropy and the evolutionary stability of plastic phenotypes: a geometric framework

**DOI:** 10.1093/g3journal/jkaf262

**Published:** 2025-11-11

**Authors:** Charles Qiujie Wang, James G DuBose

**Affiliations:** Department of Biology, Emory University, 1510 Clifton Rd NE #2006, Atlanta, GA 30322, United States; Department of Biology, Emory University, 1510 Clifton Rd NE #2006, Atlanta, GA 30322, United States

**Keywords:** pleiotropy, plasticity, geometric model

## Abstract

Phenotypic plasticity allows organisms to express different traits in response to different environmental or genetic conditions. Understanding the evolution of conditional phenotypes is challenging because they are not expressed by all members of a population, which allows for the accumulation of deleterious variation due to a reduced efficacy of purifying selection. Theory suggests pleiotropic effects help prevent the decay of conditional phenotypes by exposing the variation accrued in one context to the effects of purifying selection in an alternative context. However, existing frameworks for describing the evolutionary dynamics of conditional phenotypes are limited in their ability to flexibly model the complex pleiotropic architectures that often underlie conditional phenotypes. To help improve our understanding of the evolutionary stability of conditional phenotypes, here we describe a geometric model that allows for explicit modeling of different fitness optima for conditional and alternative phenotypes, as well as their underlying pleiotropic associations. Using stochastic simulations and mathematical analyses, we show that this model recapitulates and elaborates on existing predictions regarding the role of pleiotropy in maintaining conditional phenotypes. Specifically, we found that more pleiotropic conditional phenotypes experience decreased rates of decay in fitness over periods of inexpression, the effects of which are comparable for phenotypes that are spatially and temporally conditional. Furthermore, the functional form of the relationship between conditional phenotype expression pattern and decay rate is mediated by pleiotropic effect, which provides more explicit hypotheses of when pleiotropic constraint is expected to play a significant role in the evolutionary maintenance of conditional phenotypes. Finally, we found that when pleiotropic architectures evolve over periods of conditional phenotype inexpression, decoupling from other phenotypes readily evolves and facilitates decay in fitness.

## Introduction

Most organisms possess some capacity to express different phenotypes in response to different environmental or genetic contexts. This plasticity confers many notable ecological and evolutionary benefits, including a greater ability to invade different niches and a decreased extinction risk due to said increased niche breadth (Chevin et al. 2010; Matesanz et al. 2012; Forsman 2015). Furthermore, many taxa constitutively express traits that exist as conditionally expressed alternatives in closely related lineages, suggesting an important role of plasticity in shaping patterns of macroevolutionary diversification (Moczek et al. 2011). Although the importance of plasticity in facilitating the ecological success and evolutionary diversification of lineages has been well described, explaining the evolutionary maintenance of conditional phenotypes has been more challenging (DeWitt et al. 1998; Snell-Rood et al. 2010).

The challenge in explaining the evolutionary dynamics of conditional phenotypes lies in their frequent inexpression. Since the probability that individuals in a population will express a conditional phenotype is often low across time or space, relaxed purifying selection can allow conditional phenotypes to accrue more functional variation (Snell-Rood et al. 2010; Van Dyken and Wade 2010). For example, selection would be less effective at removing variation that is only deleterious when expressed in males because its deleterious effects are not realized when expressed in the other sex (Brisson and Nuzhdin 2008). Given the right environment, this increase in variation conferred by relaxed selective constraints could in theory facilitate rapid adaptation via positive selection (though empirical evidence is limited) (Moczek et al. 2011). However, the general and expected outcome is that variation acquired during inexpression erodes the functionality and fitness associated with conditional phenotypes (DeWitt et al. 1998; Snell-Rood et al. 2010; Van Dyken and Wade 2010; Moczek et al. 2011).

Models describing the evolutionary dynamics of conditionally expressed phenotypes typically focus on the proportion of a population that expresses said phenotype over space and time (Snell-Rood et al. 2010; Van Dyken and Wade 2010). Such frameworks implicitly consider loci as either “on” when expressed or “off” when not, which is a simplifying feature that helps make models more tractable. However, advancements in genetic sequencing and expression profiling have shown that pleiotropic effects—when a locus contributes to phenotypic production in multiple contexts—can shape evolutionary trajectories by mediating the relative importance of different evolutionary processes (Hill and Otto 2007; Mank et al. 2008; Dean and Mank 2016; Fraïsse et al. 2019; Williams et al. 2023). Therefore, frameworks that explicitly consider how pleiotropic architecture influences the balance between purifying selection and deleterious mutation accumulation may improve our understanding of the evolutionary dynamics (particularly maintenance) of conditional phenotypes (Snell-Rood et al. 2010; Moczek et al. 2011; Forsman 2015).

Existing frameworks that consider the role of pleiotropic effects in governing the evolution of conditional phenotypes typically do so by defining the selective relevance of a given variation in alternative environments (Kawecki et al. 1997; Otto 2004). In this, pleiotropy is represented from more of a “top-down” perspective, where it is considered as a selective outcome. Such approaches have been useful for studying the interplay between dispersal and antagonistic pleiotropy in constraining adaptation in alternative environments. However, pleiotropy can also be considered as an organismal property, and it has been well articulated that adopting this more “bottom-up” perspective may be more useful for describing and exploring how different pleiotropic architectures shape the evolutionary trajectories of conditional phenotypes (Forsman 2015).

To explore the potential for conditional phenotypes to be maintained via pleiotropic expression in a constitutively expressed alternative phenotype, here we describe a geometric model that considers different fitness optima associated with conditional and alternative phenotypes, which are produced through differential investments in various latent traits (Fisher 1930). This allows for explicit and flexible modeling of the pleiotropic architectures underlying conditional phenotypes at an organismal level, as well as evolving pleiotropic architectures and various patterns of expression probability. To impart greater tractability, we also analytically derive closed-form expressions that describe the decay in fitness of conditional phenotypes as a function of pleiotropic effect and expression probability. In general, we found that this model recapitulates key predictions regarding the interplay between pleiotropic architecture and the evolutionary dynamics of conditional phenotypes. However, the functional form of this interplay changes with different patterns of expression probability, thus lending more explicit hypotheses regarding the conditions in which pleiotropy is expected to be important for maintaining a conditional phenotype and when it is likely less important. Finally, pleiotropic architectures readily evolve decoupling between conditional and alternative phenotypes over periods of conditional inexpression, which facilitates decay in conditional fitness.

## Model description

The various phenotypes expressed by an organism are achieved through differential investment in various latent traits (implicitly via differential gene expression). Let $z=(z_{1},z_{2},\ldots ,z_{n})$ represent the values of these various traits. The expression of each latent trait needed to produce phenotype $P^{m}$ is given by $x^{m}=(x_{1}^{m},x_{2}^{m},\ldots ,x_{n}^{m})$, where m∈(1,2,…,M) indexes various distinct phenotypes and *M* is the number of phenotypes in consideration. In this, each *z* can be thought of as the trait value when the corresponding x=1. For example, consider an enzyme with some affinity to a substrate that creates a yellow pigment. The specific affinity of the enzyme to the substrate is the latent value *z*, while the overall yellow-ness, which is the emergent trait, will also be determined by how much the enzyme is expressed *x*. Therefore, changing yellow-ness can be accomplished by changing the enzyme’s affinity (*z*) or expression (*x*). These expression vectors then form the columns of an expression matrix $X\in R^{n\times m}$:


(1) $$X=\left[\begin{array}{cccc} x_{1}^{1} & x_{1}^{2} & \ldots & x_{1}^{m}\\ x_{2}^{1} & x_{2}^{2} & \ldots & x_{2}^{m}\\ \vdots & \vdots & \ddots & \vdots \\ x_{n}^{1} & x_{n}^{2} & \ldots & x_{n}^{m} \end{array}\right]$$

It is worth noting that this expression matrix can be thought of as analogous to what is obtained via high-throughput RNA/protein profiling. Therefore, a given phenotype $P^{m}$ is constructed by element-wise multiplication of latent traits and expression values:


(2) $$P^{m}=x^{m}\circ z.$$

The overall objective of this framework is to consider the role of pleiotropy in maintaining the traits that compose a conditional phenotype, hereafter denoted as $P^{C}$, via their phenotypic effects in an alternative constitutively expressed phenotype, hereafter denoted as $P^{A}$. Let the expression vector that constructs $P^{C}$ be $x^{C}$, and the expression vector that constructs $P^{A}$ be $x^{A}$. Note that the expression values in $x^{A}$ do not necessarily have to represent those from a single alternative phenotype. Rather, they may represent expression values from various phenotypes. Since this is fundamentally a model of stabilizing selection, it is most useful to consider the alternative contexts in which the strongest purifying selection is exerted. This can be readily inferred via expression levels, as there is an inverse relationship between expression level and evolutionary rate (Subramanian and Kumar 2004; Drummond et al. 2005; Cherry 2010; Dasmeh and Serohijos 2017; Trucchi et al. 2024). Therefore, $x^{A}$ can be considered as the phenotype in which it is maximally expressed, where the expression of each trait in the alternative context is $x_{n}^{A}=\max_{m\neq C}x_{n}^{m}$. Given these expression vectors, the degree of pleiotropy between $P^{C}$ and $P^{A}$ for trait $z_{n}$ can be evaluated as its relative expression in $P^{C}$:


(3) $$r_{n}=\;\frac{x_{n}^{C}}{x_{n}^{A}+x_{n}^{C}}$$

Therefore, the breadth of pleiotropic influence of each trait is then $r=(r_{1},r_{2},\ldots ,r_{n})\in [0,1]$, where


$$\begin{array}{rl} r_{n} & =1\;:\;\;\text{trait only contributes to}\;P^{C},\\ r_{n} & =0\;:\;\;\text{trait only contributes to}\;P^{A},\\ r_{n} & =0.5\;:\;\;\text{trait contributes equally to}\;P^{C}\;\text{and}\;P^{A} \end{array}$$

Since *r* maintains the relative differences between $P^{C}$ and $P^{A}$, each phenotype can be defined in terms of *r*, where $P^{C}$ is


(4) $$P^{C}=r\circ z$$

and $P^{A}$ is


(5) $$P^{A}=(1-r)\circ z$$

**Fig. 1.**
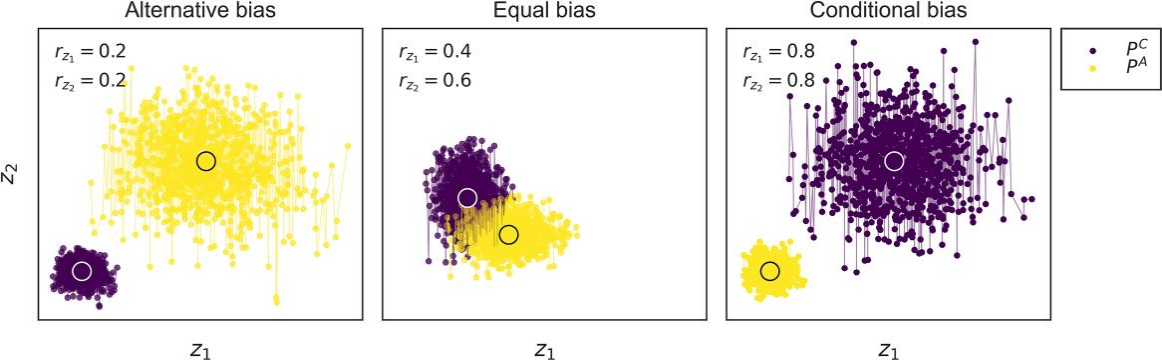
The influence of *r* on how mutations perturb conditional and alternative phenotypes. Each panel depicts the changes in each mean phenotype when evolved under high mutational effect and weak stabilizing selection (for clearer depiction). In each panel, the x and y axes represent the trait values for traits $z_{1}$ and $z_{2}$, respectively, where their corresponding *r* values are in the upper left. Each point represents the mean trait value for the population, and lines connecting points depict their evolutionary trajectories. The larger circles at the center of each temporal phenotype distribution represents the location of the corresponding phenotypic optimum. In the leftmost panel, both *r* values are small (<0.5), indicating that the traits are mostly expressed in $P^{A}$. Therefore, mutation significantly impacts $P^{A}$ and has notably less impact on $P^{C}$. In the middle panel, the *r* values for each trait are close to 0.5, indicating they are expressed to similar extents in $P^{C}$ and $P^{A}$. Therefore, mutation impacts both phenotypes to similar extents. In the rightmost panel, both *r* values are large (>0.5), indicating that the traits are mostly expressed in $P^{C}$. Therefore, mutation significantly impacts $P^{C}$ and has notably less effect on $P^{A}$.

To provide a more intuitive sense of how *r* influences $P^{C}$ and $P^{A}$, Fig. 1 depicts the evolution of each phenotype with varying degrees of *r*. While this approach removes the magnitude of investment in each trait, it conserves the general property where more context-specific traits tend to contribute proportionally less to other contexts, an assumption that is empirically supported and commonly made (Guillaume and Otto 2012). Furthermore, it conserves the behavior of mutational perturbation, and provides a continuous modulator of pleiotropic association between phenotypes.

The fitness associated with expression of $P^{C}$ and $P^{A}$ depends on the optimal phenotypes in their respective contexts, which are described by the vectors $o^{C}=(o_{1}^{C},o_{2}^{C},\ldots ,o_{n}^{C})$ and $o^{A}=(o_{1}^{A},o_{2}^{A},\ldots ,o_{n}^{A})$, respectively. Note that the optimal value for each trait is determined by both *z* and *r*:


(6) $$o_{n}^{C}=r_{n}^{o}\cdot z_{n}^{o}\;\text{and}\;o_{n}^{A}=(1-r_{n}^{o})\cdot z_{n}^{o}$$

where $r_{n}^{o}$ represents the optimal investment in trait $z_{n}^{o}$ for $P^{C}$ relative to $P^{A}$, as previously described. Therefore, the fitness associated with $P^{C}$ is


(7) $$W^{C}=\exp\;(-\sigma_{s}\|P^{C}-o^{C}{\|}^{2})$$

and the fitness associated with $P^{A}$ is


(8) $$W^{A}=\exp\;(-\sigma_{s}\|P^{A}-o^{A}{\|}^{2})$$

where $\|P^{G}-o^{G}{\|}^{2}$ and $\|P^{C}-o^{C}{\|}^{2}$ are the Euclidean distances between each phenotype and its corresponding optimum, and $\sigma_{s}$ is the strength of stabilizing selection. The important factor for examining the evolutionary stability of a conditional phenotype is expected fitness, given the probability that individuals will express the conditional phenotype. Let $f_{C}$ and $f_{A}$ be the probabilities that an individual will express the conditional and alternative phenotypes, respectively. Since our goal is to consider the extent to which conditional phenotypes can be maintained via a constitutively expressed alternative phenotype, $f_{A}=1$. Therefore, there is no fitness consideration for if $P^{A}$ is not expressed. Likewise, the contribution of non-expressed phenotypes to fitness is either zero or constant and therefore do not impact relative fitness. Assuming additive fitness for simplicity, the expected total fitness $W^{T}$ for an individual is given in Equation 9 (see Supplementary Section 1.1 for full derivation).


(9) $$W^{T}=W^{C}\cdot f_{C}+W^{A}\cdot f_{A}$$

Mutations occur that are drawn from a normal distribution to make small random changes to the trait vector *z*, which affects the phenotype in an additive fashion. Let $\sigma_{m}$ be the standard deviation in mutational effects. The mutational effect vector is then $\Delta z=(\delta_{1},\delta_{2},\ldots ,\delta_{n})$ where $\delta_{i}∼N(0,\sigma_{m})$. Therefore, the trait vector for a mutated individual is $z^{\prime }=z+\Delta z$, which has the potential to influence $P^{C}$ and $P^{A}$ because they both depend on *z*. *r* can be mutated in a similar fashion. Let $\sigma_{r}$ be the standard deviation in mutational effects on *r*. The *r* mutation effect vector is then $\Delta r=(\rho_{1},\rho_{2},\ldots ,\rho_{n})$, where $\rho_{n}∼N(0,\sigma_{r})$. Therefore, the *r* vector for a mutated individual is $r^{\prime }=\mathrm{clip}(r+\Delta r,0,1)$, where the clip function ensures $r^{\prime }\in [0,1]$. Mutant phenotypes for $P^{C}$ and $P^{A}$ can then be described as:


(10) $$P^{C^{\prime }}=r^{\prime }\circ z^{\prime }\;\text{and}\;P^{A^{\prime }}=(1-r^{\prime })\circ z^{\prime }$$

respectively. It is worth noting that this approach is not sensitive to the value of a trait nor to the direction of mutational perturbation. The only factor considered in determining fitness is the distance of the trait from its optimum. This flexibility accommodates biological systems well, as decreasing the value of a trait may be what defines adaptive relevance or an alternative phenotype.

## Methods

### Definition of pleiotropic architectures

The distribution of *r* describes the overall pleiotropic architecture of a conditional phenotype, which can be flexibly represented using a *β*-distribution, such that $r_{n}∼\text{Beta}(\alpha ,\beta )$. The *β*-distribution is a desirable choice because it is naturally bound between 0 and 1, and the magnitude of *α* and *β* determine how concentrated the distribution is around the mean, which is $\mu =\frac{\alpha }{\alpha +\beta }$. Therefore, if α=β, greater values of both parameters represent more pleiotropic architectures because traits tend to be concentrated around the mean of r=0.5.

### Evolutionary simulations

For simplicity, we considered a haploid asexual population and performed evolutionary simulations using a standard Wright-Fisher approach. That is, let *N* be the population size. To create each new generation, *N* parental phenotypes are first selected with replacement, where selection probabilities are proportional to relative fitness. Selected parental phenotypes are then mutated as previously described to create the phenotypes of individuals in the next generation. Simulations presented in this study were conducted with the following parameters: μ=0, $\sigma_{m}=0.1$, $\sigma_{s}=1$, $N_{e}=1000$, n=20, and $z_{0}=(1,1,\ldots ,1)$. Specification of other parameter values are given in the simulation description.

### Closed-form expressions describing conditional fitness decay rate

Although the probabilistic nature of the the previously described model may represent more realistic uncertainty, it suffers from intractability. Therefore, to better discern the interplay between pleiotropic effect and expression probability in mediating the decay of conditional phenotypes, we used the previously described framework to derive a closed-form expression of this relationship. Since our primary objective was to describe the evolutionary stability of a conditional phenotype as a function of its pleiotropic links to a constitutively expressed alternative phenotype, we assume $f_{A}=1$ (see Supplementary Section 1.2 for a more general description). Therefore, we approximate $W^{T}$ by a single Gaussian weight $\exp\;[-S\;x^{2}]$, where


(11) $$S=\sigma_{s}\frac{\;f_{C}\;r^{2}+(1-r{)}^{2}}{1+f_{C}}$$

and *x* is the deviation of the trait from the optimum. This ansatz follows from the second-order Taylor expansion of $\ln\;W^{T}(x)$ around (x=0), and is exact up to quadratic order in *x*. From there, we assume that after mutation in generation *t*, the population-level trait distribution is $z∼N(z_{0},V_{t})$, a normal distribution with optimum $z_{0}$ and variance $V_{t}$. We then derive an exact recursion for $V_{t}$ by re-weighting the prior


(12) $$p_{t}(z)=\frac{1}{\sqrt{2\pi V_{t}}}\exp\;\left[-\frac{(z-z_{0}{)}^{2}}{2V_{t}}\right]$$

with $W^{T}(z)\approx \exp\;[-S\;(z-z_{0}{)}^{2}]$. This gives the unnormalized posterior


(13) $$p_{t}^{\prime }(z)\propto \exp\;\left[-\left(S+\frac{1}{2V_{t}}\right)(z-z_{0}{)}^{2}\right]$$

Therefore, exactly after selection, the variance is


(14) $$V_{t}^{\prime }=\frac{1}{2\;(S+1/(2V_{t}))}=\frac{V_{t}}{1+2\;S\;V_{t}}$$

Because mutation adds $\sigma_{m}^{2}$ to the variance, we then convolve the two Gaussian functions to the exact difference equation of $V_{t}$


(15) $$V_{t+1}=\frac{V_{t}}{1+2\;S\;V_{t}}+\sigma_{m}^{2},$$

Given $z∼N(z_{0},\;V_{t})$, conditional phenotype fitness can be considered $W^{C}(z)=\exp\;[-\sigma_{s}\;r^{2}\;(z-z_{0}{)}^{2}]$, whose Gaussian integral is


(16) $$E\left[W^{C}(t)\right]=\int e^{-\sigma_{s}r^{2}(z-z_{0}{)}^{2}}\frac{1}{\sqrt{2\pi V_{t}}}e^{-\frac{(z-z_{0}{)}^{2}}{2V_{t}}}\;dz={\left[1+2\;\sigma_{s}\;r^{2}\;V_{t}\right]}^{-1/2}$$

By definition, the decay rate is $\lambda_{C}(f_{C},r)=-\frac{1}{t}\ln\;(\frac{E[W_{C}(t)]}{W_{C}(0)})$. Since under the initial condition $V_{0}=0\Rightarrow E[W_{C}(0)]=1$, the closed-form is


(17) $$\lambda_{C}(f_{C},r)=\frac{1}{2\;t}\ln\;\left[1+2\;\sigma_{s}\;r^{2}\;V_{t}\right]$$

This equation now allows us to run a direct numerical iteration for the exact recursion.

Similarly, we considered temporal variation in conditional phenotype expression and and derived a deterministic expression of generations between expression *g* of the conditional phenotype, defining the update maps:


(18) $$F^{A}(V)=\frac{V}{1+2\;S^{A}\;V}+\sigma_{m}^{2},\;F^{C}(V)=\frac{V}{1+2\;S^{C}\;V}+\sigma_{m}^{2}$$

where $S^{A}=\sigma_{s}(1-r{)}^{2}$ and $S^{C}=\sigma_{s}\;r^{2}$. By definition, the on-off cycle of *g* is completed starting from $V_{0}=0$, applying $F^{A}$  (g−1) times, and $F^{C}$ once. The variance at steady state is described by the fixed-point equation


(19) $$V^{*}=F_{C}\left(F_{A}^{\circ (g-1)}(V^{*})\right)$$

which is a contraction on [0,∞), and converges uniquely by iterating from $V_{0}=0$. Knowing that just before the on-step, the variance is $V_{\left(g-1\right)}$, the fitness at the on-step is described exactly


(20) $$E[W_{C}{]}_{\mathrm{burst}}=\int e^{-S^{C}(x-z_{0}{)}^{2}}\frac{e^{-(x-z_{0}{)}^{2}/(2V^{\left(g-1\right)})}}{\sqrt{2\pi \;V^{\left(g-1\right)}}}\;dx=[1+2\;S^{C}\;V^{\left(g-1\right)}{]}^{-1/2}$$

Distributing the single-cycle log loss evenly over *g* generations for interpretation free from in-cycle fluctuation, we get


(21) $$\lambda_{C}(r,g)=-\frac{1}{g}\ln\;(E[W_{C}{]}_{\mathrm{burst}})=\frac{1}{2g}\ln\;[1+2\;\sigma_{s}\;r^{2}\;V^{\left(g-1\right)}]$$

Because there is exactly one on-step per *g* generations, we have


(22) $$f_{C}=\frac{1}{g},\;g=\frac{1}{f_{C}},\;f_{C}\in \left\{1,\frac{1}{2},\frac{1}{3},\ldots \right\}$$

which is biologically defined only for all g>1.

### Analysis of evolving pleiotropic architectures during periods of conditional phenotype inexpression

To begin exploring the consequences of an evolving pleiotropic architecture on the fitness associated with conditional phenotypes, we first conducted evolutionary simulations as previously described. However, in addition to simulations where $\sigma_{r}=0$ (non-evolving *r*), we also conducted simulations where $\sigma_{r}=0.01$. Otherwise, all parameters were set as previously described.

When conditional phenotypes are not expressed, evolutionary dynamics (in both *z* and *r*) are governed by the fitness landscape of alternative phenotypes. Therefore, to begin understanding how pleiotropic architectures are expected to evolve during periods of conditional phenotype inexpression, we analyzed how different alternative optima shape the fitness landscapes across the *z* and *r* parameter space. First, we assume a uniform trait density on a fixed window $z\in [z_{\min},z_{\max}]$. For the purposes of our analyses, $\left[z_{\min},z_{\max}]=[-3,3\right]$. The fitness landscape for alternative phenotypes for a given *r* is


(23) $$I(r)=\int_{z_{\min}}^{z_{\max}}W^{A}(r,z)\;dz$$

Differentiating Equation 23 for I(r) with respect to *r* yields the marginal selection gradient:


(24) $$\frac{dI}{dr}(r)=\frac{1}{a}\left[I(r)+z_{\min}\exp\;\left(-u_{\min}^{2}\right)-z_{\max}\exp\;\left(-u_{\max}^{2}\right)\right].$$

Under a symmetrical window, the boundary limit r→1 exists, where $I^{\prime }(1)=0$. The full derivation can be found in Supplementary Section 1.3.

### Simulations and numerical analyses

To confirm the relative importance of pleiotropic effect (*r*) and expression frequency ($f_{C}$) in maintaining the fitness of conditional phenotypes suggested by our deterministic model, we leveraged the exponential nature of fitness decay to numerically estimate the decay rate of conditional phenotype fitness. Specifically, the fitness of a conditional phenotype ($W^{C}$) exponentially decays with respect to the Euclidean distance from the optimum. As mutations accumulate, the distance between $P^{C}$ and $o^{C}$ is expected to increase linearly on average. Therefore, the expected decay in fitness associated with a conditional phenotype can be expressed as:


(25) $$E[W_{t}^{C}]\approx (W_{0}^{C})\cdot \exp^{-\lambda_{C}t}$$

where $\lambda_{C}$ is the decay rate. To parallel the predictions from our analytic solutions, we focused on evaluating the fitness decay of single conditional traits. This allowed for tractable simulation of the evolutionary trajectories across a range of *r* and $f_{C}$ values, which could then be used estimate the decay in conditional fitness $\lambda_{C}$ by fitting Equation (25) to the resulting dynamics. To reduce the error associated with stochastic mutation and probabilistic sampling, we ran each simulation 10 times and fit Equation (25) to the average dynamics. Furthermore, we used Gaussian smoothing to generate a smoother contour surface of changes in $\lambda^{C}$ across this parameter space. We performed all simulations using Python (available as supplemental information), and we used the *SciPy* Python library for all model fitting and Gaussian smoothing (Virtanen et al. 2020).

We used a similar approach evaluate our analytical predictions of how how pleiotropy mediates the effect of temporal variation in expression frequency on conditional phenotype fitness decay. Here, we defined expression regimes that varied in the number of generations between expression of the conditional phenotype (*g*):


(26) $$f_{C}(i;g)=\left\{ \begin{array}{cc} 1, & \text{if}\;i\mathrm{mod}g=0\\ 0, & \text{otherwise}\; \end{array}\right.$$

where *i* is the generation index and $f_{C}=1$ every *g* generations. We then simulated evolutionary dynamics across the *r* and *g* parameter space and estimated conditional fitness decay ($\lambda_{C}$) as previously described.

## Results

### More pleiotropic genetic architectures maintain greater fitness associated with conditional phenotypes during prolonged periods of inexpression

**Fig. 2.**
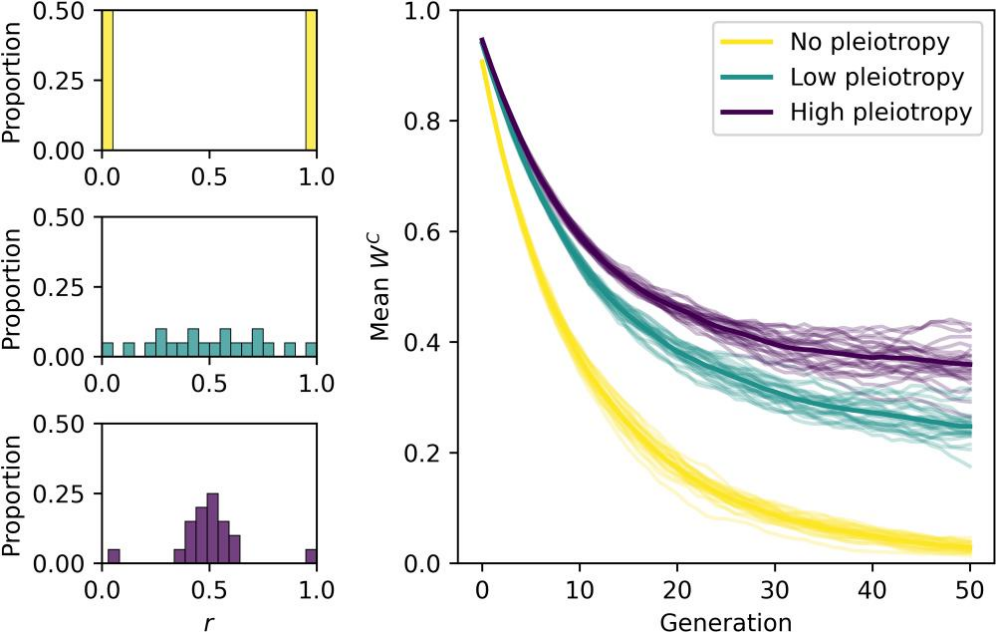
More pleiotropic architectures allow for greater maintenance of conditional phenotype fitness when not expressed ($f_{C}=0$). Histograms in the leftmost panels show different pleiotropic architectures (distributions of *r*) that may underlie a conditional phenotype. The upper panel depicts a non-pleiotropic architecture, the middle panel depicts a low-pleiotropic architecture, and the bottom panel depicts a highly pleiotropic architecture. The panel on the right shows the change in average fitness associated with a conditional phenotype (mean $W^{C}$) while the population evolves without expressing it. Each smaller and lighter line represents the dynamics from independent simulations, and the larger and darker lines represent the average dynamics across simulation.

Although general theory suggests that pleiotropy should constrain the divergence of conditional phenotypes, consideration of said theory primarily focuses on pleiotropic constraints on positive selection. However, the same concept should be applicable to purifying selection as well. To explore this concept with the previously described model, we simulated the evolutionary dynamics of three conditional phenotypes that were not expressed ($f_{C}=0$) for fifty generations with different underlying pleiotropic architectures. First, we considered a “no pleiotropy” architecture, where all traits were either totally expressed in the conditional phenotype (r=1) or totally expressed in the alternative phenotype (r=0). We then considered a “low pleiotropy” architecture by defining the distribution of *r* using a Beta distribution where α=2 and β=2, which creates a somewhat uniform distribution. Finally, we considered a “high pleiotropy” architecture by defining the distribution of *r* using α=20 and β=20, which creates a distribution where most traits are concentrated near r=0.5 and with few traits near r=0 or r=1 (Fig. 2).

Consistent with expectations, the fitness associated with the non-pleiotropic conditional phenotype deteriorated rapidly (Fig. 2). However, even a low degree of pleiotropy significantly decreased the rate at which conditional phenotypes decayed in fitness (Fig. 2). Likewise, a high degree of pleiotropic coupling decreased this decay rate even more throughout the period of inexpression (Fig. 2).

### The significance of pleiotropic constraint in maintaining conditional traits is greatest when infrequently expressed and deteriorates with increasing probability of expression

**Fig. 3.**
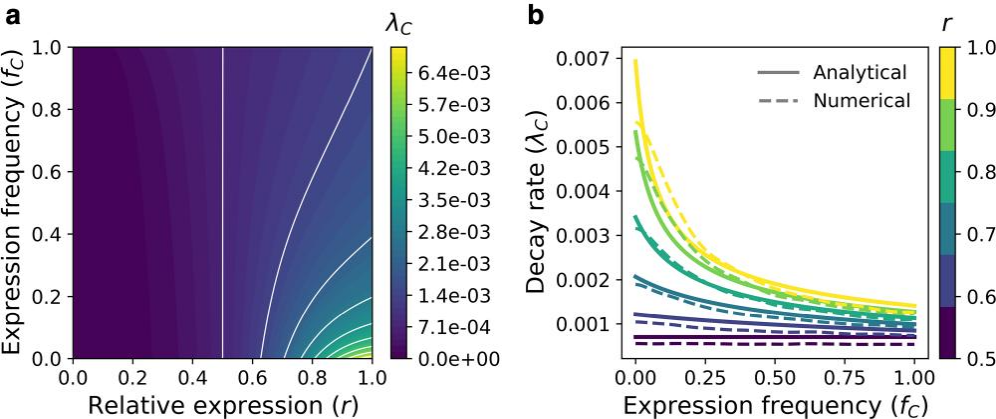
The role of pleiotropic constraint in shaping the fitness decay of conditional traits. a) A contour plot showing decay rate ($\lambda_{C}$) as a function of relative expression in the conditional phenotype (*r*) and expression probability ($f_{C}$). Lighter colors indicate greater decay rates, which occur when expression probability is lower and relative expression is higher. b) Decay rate in conditional phenotype fitness as a function of expression probability for traits with varying degrees of pleiotropy (*r*). Lighter colors represent higher values of *r* and therefore, less pleiotropy. Solid lines represent analytical solutions, while dashed lines represent numerically estimated decay rates from simulations. Taken together, A and B illustrate how pleiotropic effects mediate the rate at which conditional phenotypes decay with changing expression probability.

To explore the relative importance of expression probability ($f_{C}$) and pleiotropy (*r*) in preventing the decay in fitness associated with conditional traits ($\lambda_{C}$), we used the previously derived closed-form expression (Equation 17) to examine the rate of fitness decay across the $f_{C}$–*r* parameter space (for single conditional traits). Consistent with expectations, conditional traits with little pleiotropic effect (r≈1) and low expression probability ($f_{C}\approx 0$) exhibited the highest decay rates (Fig. 3a). Conversely, increasing either expression probability or pleiotropic effect (reducing r→0.5) decreases decay rate, though the extent of this decrease was shaped by their interaction (Fig. 3a). To better illustrate this pattern, we visualized the decay rate as a function of expression probability across different degrees of pleiotropic effect (0.5<r≤1). This showed the reduction in decay rate associated with increasing expression probability was most pronounced in weakly pleiotropic traits (r≈1), a pattern that diminished with increasing pleiotropic effect (r→0.5). In other words, the rate at which $\lambda_{C}$ declined with respect to $f_{C}$ decreased with greater pleiotropic effect (r→0.5). We further confirmed that these predictions were consistent with our stochastic model by conducting simulations based on single traits across the *r* and $f_{C}$ parameter space and numerically estimating decay rates (Fig. 3, Supplementary Section 2.1, Supplementary Fig. S1). Taken together, these findings lend support to the prediction that the importance of pleiotropic constraint in preventing the decay of conditional traits may be significant when rarely expressed but rapidly diminishes with increasing expression probability.

### The role of pleiotropic constraint in maintaining conditional phenotypes is comparable between traits with spatially and temporally conditional expression

**Fig. 4.**
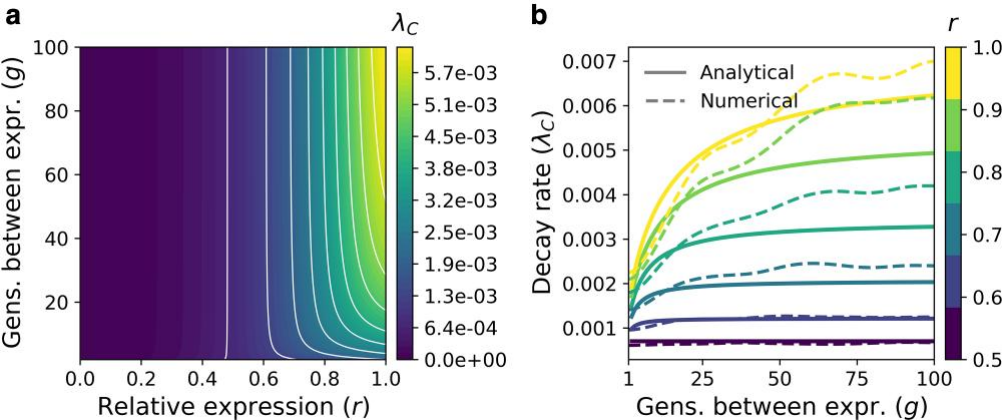
The role of pleiotropic constraint in shaping the fitness decay of traits that are temporally conditional. a) A contour plot showing variation in decay rate ($\lambda_{C}$) as a function of relative expression in the conditional phenotype (*r*) and generations between expression (*g*). Lighter colors indicate greater decay rates, which occur when relative expression is higher and there are more generations between expression. b) Decay rate in conditional phenotype fitness as a function of generations between expression for traits with varying degrees of pleiotropy (*r*). Lighter colors represent higher values of *r* and therefore, less pleiotropy. Solid lines represent analytical solutions, while dashed lines represent numerically estimated decay rates from simulations. Note that slight deviations in numerical estimates are expected because simulated dynamics have temporal cycles that increase in magnitude with higher *r*. Taken together, A and B illustrate how pleiotropic effects mediate the rate at which conditional phenotypes decay with changing temporal expression probability, which is consistent with patterns of decay for spatially conditional phenotypes.

The previous analysis implicitly focused on traits that are conditionally expressed spatially, where $f_{C}$ is determined by how frequently an individual within a population encounters the cuing environment or genetic background. However, conditional expression also occurs temporally, where all or most individuals in a population express a trait every several or many generations. To examine the interplay between pleiotropic constraint and fluctuating temporal expression probability, we conducted the previously described analysis but instead specified the number of generations (*g*) between expression (where $f_{C}=1$ and $f_{A}=0$ when expressed). Similar to patterns in traits with conditional spatial expression, conditional traits that go many generations without expression experience little pleiotropic constraint (r⪆0.9) and exhibit the highest rates of fitness decay ($\lambda_{C}$) (Fig. 4a). Likewise, both reducing the number of generations between expression and increasing pleiotropic effect (reducing r→0.5) decreases the decay rate with some degree of interaction (Fig. 4a). Examining decay rate as a function of generations between expression across various degrees of pleiotropy (0.5<r≤1) showed that the increase in decay rate associated with more generations between expression was most pronounced in weakly pleiotropic traits and declined with increasing pleiotropic effect (r→0.5) (Fig. 4b). We confirmed that these predictions were consistent with our stochastic model by conducting simulations based on single traits across the *r* and *g* parameter space and numerically estimating decay rates (Fig. 4, Supplementary Section 2.1, Supplementary Fig. S2). These findings echo those of traits with spatially conditional expression, suggesting comparable effects of pleiotropic constraint.

### Patterns and consequences of directional and stabilizing selection on evolving pleiotropic architectures over periods of conditional inexpression

**Fig. 5.**
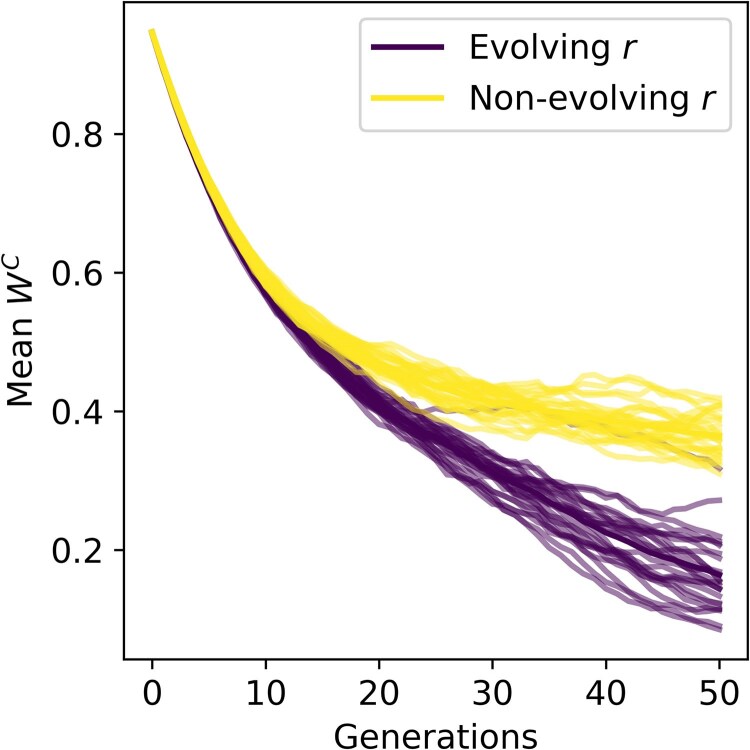
Evolving pleiotropic architectures facilitate the decay in fitness associated with conditional phenotypes when not expressed. Each line represents an independent simulation, which shows the change in conditional phenotype fitness (*y*-axis) over generations of evolution (*x*-axis). Darker lines depict simulations where pleiotropic architecture was allowed to evolve by setting $\sigma_{r}=0.01$, while the lighter lines depict simulations where $\sigma_{r}=0$ (no evolution). The darker line depicted with each simulation type represents the average dynamics across replicate simulations. Overall, the dynamics show that when *r* distributions are allowed to evolve, the conditional fitness decays more rapidly.

Our previous analyses focus on the evolutionary dynamics of conditional phenotypes given a stable pleiotropic architecture (*r* distributions). However, pleiotropic associations can evolve as well. To begin exploring the consequences of evolving *r* distributions, we first conducted simulations as previously described but allowed *r* to mutate and evolve along with *z*. This showed that an evolving pleiotropic architecture increases the decay in fitness associated with conditional phenotypes when not expressed (Fig. 5).

**Fig. 6.**
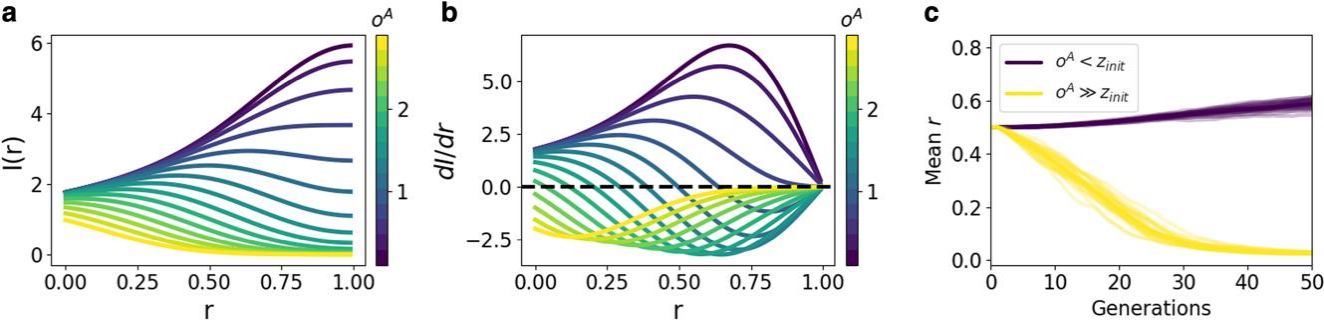
Fitness landscapes associated with different alternative optima predicts directional selection on pleiotropic architecture. a) The integrated alternative-phenotype fitness as a function of varying degrees of pleiotropy (*r*). Different lines represent different alternative phenotype optima $o^{A}$, spanning (0,3), where lighter colored lines represent $o^{A}$ values that are more distant from the initial z value. Note that functions are symmetric around $o^{A}=z_{\mathrm{init}}$, as what matters is the absolute difference between $o^{A}$ and $z_{\mathrm{init}}$. b) The marginal selection gradient $\frac{dI}{dr}$ as a function of (*r*), where lines correspond to those in panel A. Taken together, A and B illustrate the expected direction of change in *r*, which depends on the sign of $\frac{dI}{dr}$. Since the sign of $\frac{dI}{dr}$ is determined by the value of $o^{A}$ relative to $z_{\mathrm{init}}$, there are three scenarios for change. First, if $o^{A}\ll z_{\mathrm{init}}$, $\frac{dI}{dr}>0$ and therefore *r* increases. Second, if $o^{A}\approx z_{\mathrm{init}}$, *r* stabilizes where $\frac{dI}{dr}=0$. Finally, if $o^{A}\gg z_{\mathrm{init}}$, $\frac{dI}{dr}<0$ and therefore *r* decreases. C) Evolutionary simulations are consistent with the dynamics predicted in panels A and B. Here, the *y*-axis shows the mean degree of pleiotropy (*r*), and the *x*-axis represents generations. Darker lines depict simulations where $0<o^{A}<z_{init}$, a value less than initial latent value $z_{init}=1$. Lighter lines depict simulations where $o^{A}\gg z_{init}$, a value much larger than initial latent value $z_{init}=1$. Taken together, these predictions and simulations suggest the dynamics of pleiotropic decoupling during periods of conditional phenotype inexpression depend on the alternative phenotype’s fitness landscape.

To gain further insight into these fitness dynamics, we then examined how pleiotropic architectures evolve over periods of conditional inexpression. Evolutionary dynamics, in both trait values (*z*) and relative expression values (*r*), are governed by the fitness landscape of alternative phenotypes when conditional phenotypes are not expressed. Therefore, to predict how pleiotropic associations *r* evolve, we examined how different alternative optima $o^{A}$ shape fitness landscape (integrated fitness functions for a given *r*) of alternative phenotypes (Fig. 6a and b). This revealed three scenarios that depend on the initial deviation of trait values from $o^{A}$. First, if $o^{A}\ll z_{\mathrm{init}}$, the change in fitness associated with increasing *r* is always positive. Therefore, *r* is expected to increase (Fig. 6b). Likewise, if $o^{A}\gg z_{\mathrm{init}}$, the change in fitness associated with increasing *r* is always negative, and *r* is therefore expected to decrease (Fig. 6b). Finally, if $o^{A}\approx z_{\mathrm{init}}$, there exists some equilibrium fitness where *r* is expected to stabilize (Fig. 6b). To confirm these predictions, we then conducted simulations under scenarios where alternative optima were $o^{A}<z_{\mathrm{init}}$ and $o^{A}\gg z_{\mathrm{init}}$. Here, we set $\sigma_{r}=0.01$, and defined the $o^{A}\gg z_{\mathrm{init}}$ scenario by multiplying the defined alternative optima by 8 (to mitigate floating point operation issues). Consistent with predictions, this showed that when $o^{A}<z_{\mathrm{init}}$, pleiotropic associations *r* tended to increase (Fig. 6c). Likewise, when $o^{A}\gg z_{\mathrm{init}}$, *r* tended to decrease (Fig. 6c). Taken together, these findings illustrate the conditions in which pleiotropic associations may be maintained by stabilizing selection or pushed towards greater decoupling by directional selection.

## Discussion

To improve our understanding of the interplay between pleiotropy and expression pattern in shaping the evolutionary stability of conditional phenotypes, here we described a geometric model that allows for explicit specification of pleiotropic architecture and population expression dynamics. Consistent with previously articulated theory, we found that greater stability (maintenance of higher fitness over time) of conditional phenotypes was facilitated by more pleiotropic architectures (Fig. 2). We then analytically derived closed-form expressions describing these dynamics for individual traits, which showed that pleiotropic effects play equivalent roles in mediating the decay in fitness of phenotypes that are spatially and temporally conditional (Figs. 3, 4). These findings suggest this model provides a sufficient quantitative description of previously articulated theory. We then used this model to explore how the patterns and consequences of pleiotropic evolution, which showed that evolving pleiotropic architectures facilitate the decay of conditional phenotypes when not expressed (Fig. 5). Using analytical and simulation-based analyses, we then described how the the fitness landscape of alternative phenotypes determines the conditions in which selection favors pleiotropic decoupling versus pleiotropic stability (Fig. 6).

### Assumptions and limitations

Our choice of a geometric framework is motivated by the argument that plasticity and pleiotropy represent organismal properties that should be evaluated from a whole-organism perspective, rather than a single-trait or single-allele perspective often adopted by existing population genetic and quantitative genetic frameworks (Lande 1980; Wagner and Zhang 2011; Forsman 2015; Sultan 2021). Philosophically, a geometric framework is the most consistent with this perspective. However, the geometric framework is not without criticism or controversy, the most prominent of which is the common association between geometric models and the assumption of universal pleiotropy, where every gene or mutation affects every trait. This is potentially problematic in typical geometric frameworks because it is not possible to statistically quantify the full pleiotropic extent of a mutation and therefore, the assumption of universal pleiotropy is not empirically falsifiable (Wagner and Zhang 2012; Zhang 2023). However, in the formulation we have described here, mutational effects are confined to single-trait dimensions and pleiotropic effects between phenotypes are explicitly modeled. While this approach alleviates concerns regarding the validity of universal pleiotropy, it also removes pleiotropic associations that occur within conditional and alternative phenotypes independent of their associations with each other. This assumption is not realistic, but the objective of this study was to evaluate the potential for pleiotropic associations with alternative phenotypes to maintain conditional phenotypes, rather than to evaluate the role of within-phenotype pleiotropy. Therefore, this abstraction helps create a clearer description of the subject of study, but inclusion of complex gene regulatory networks may capture more realistic complexity (Wagner and Zhang 2011; Sakamoto and Innan 2024).

Several assumptions and simplifications regarding how pleiotropic architectures are defined and how they mutate in this model warrant additional comments as well. The first of which is the use of relative expression values to define phenotypic positions within the multi-dimensional trait space. This simplification aids in tractability but removes expression magnitude, which may influence the expected distribution of mutational effect sizes for different genes and traits (Drummond et al. 2005; Serohijos et al. 2012; Dasmeh and Serohijos 2017). However, distributions of mutational effect sizes may also vary due to many other genetic and environmental factors, the inclusion of which would significantly reduce model tractability (Dittmar et al. 2016). Furthermore, there are several lines of evidence that make this assumption defensible, at least given our current understanding of mutational effect size distributions. First, there are many empirical examples of variation in quantitative traits being explained by the cumulative effect of small-effect mutations that have been defined regardless of expression level (Yang et al. 2010; Dittmar et al. 2016; Boyle et al. 2017; Hua and Springer 2018). While the same can of course be said for fewer large-effect mutations, this suggests the assumed common distribution of small mutational effects is satisfied in at least some contexts. Second, there is an empirically well-defined inverse relationship between expression level and context specificity (Zhang and Li 2004; Yanai et al. 2005; Dean and Mank 2016; Kryuchkova-Mostacci and Robinson-Rechavi 2016). In other words, the genes or traits that differentiate a conditional phenotype from alternative phenotypes are expected to be lowly expressed. Therefore, even if expression level indeed influences mutational effect size in some contexts, this model would predominately be dealing with classes of genes or traits that are lowly expressed and would therefore have similar expected mutational effect size distributions.

Current empirical understanding of how patterns of gene expression mutate and evolve offers relatively less justification for the assumed mutational effect size distribution for relative expression vectors (*r* distributions), as experimental and inferential studies have come to a broad range of conclusions (Gilad et al. 2008). However, frequently observed distributions of small-effect regulatory mutations suggest this assumption is satisfied in at least some empirical contexts (Gruber et al. 2012; Vernot et al. 2012; Smith et al. 2013; Murphy et al. 2023). The inferences that stand to be the most affected by assumptions of regulatory mutational effects are those regarding the evolution of pleiotropic decoupling during periods of conditional phenotype inexpression. However, this model suggests such decoupling is explainable by the fitness landscape associated with alternative phenotypes (Fig. 6). Therefore, different assumptions of regulatory mutations may impact the rate of these dynamics, but are unlikely to impact the inference as a whole.

For simplicity, we also assume additive fitness, thus removing potential contributions from epistatic effects. However, inclusion of epistatic effects has the potential to alter several of the patterns we described. For example, antagonistic epistasis would accelerate decay rates because expression in both contexts would impose a greater fitness cost not captured by additive fitness. Likewise, synergistic epistasis would make expression in both contexts more favorable, thus reducing decay rates beyond predictions. Overall, epistatic effects would likely introduce distortions in the decay rate dynamics we presented here, an issue that would be more prominent in conditional and alternative phenotypes that were composed of less modular genetic architectures. Nonetheless, assuming additivity allowed us to more clearly isolate the effects of conditional expression pattern and pleiotropy. However, future investigations of how epistatic effects influence the patterns presented here would be useful for fully understanding their role in the decay of conditional phenotypes.

Finally, this model does not consider population dynamics and therefore excludes potential effects of demography, density, mutation rate, and dispersal/population structure. These features were excluded to more clearly discern pleiotropic effects that might have a lower relative importance in governing the evolution of conditional phenotypes than population dynamics. For example, one would expect that in a small population, even if pleiotropy could expose variation that would erode a conditional phenotype to purifying selection via its effects on alternative phenotypes, the efficacy of selection in general may be overridden by high susceptibility to drift. This would effectively diminish the contribution of pleiotropy towards maintaining a conditional phenotype. On a similar note, our model considers the balance between mutation and purifying selection but does not explicitly model drift, which is also hypothesized to contribute the decay of unexpressed conditional phenotypes (Van Dyken and Wade 2010). Therefore, population dynamics are undoubtedly important to consider and account for when empirically studying the efficacy of pleiotropic constraint and the erosion of conditional phenotypes. However, said dynamics predictably mediate the relative importance of fundamental evolutionary processes and were therefore not the subject of this investigation.

### Situation within existing theory and frameworks

The approach we have proposed here draws inspiration from two primary types of previously described models. First, population genetic models have been previously used to describe how conditional gene expression shapes the frequencies and fixation probabilities of genetic variants within a population (Snell-Rood et al. 2010; Van Dyken and Wade 2010). Such models have provided predictions of how the frequency at which a conditional phenotype is expressed through time and space in a population shapes patterns of genetic variation and divergence. However, incorporation of pleiotropy in this type of model has been predominately limited to considering pleiotropic effects as outcomes of selection across a heterogeneous environment, rather than an organismal property (Kawecki et al. 1997; Otto 2004). In contrast, the model we have described here takes more of a “bottom-up” perspective, where pleiotropic effects are an emergent property of differential investment in shared traits. Second, quantitative genetic models of mutation-selection balance, particularly for multivariate traits, have well described how pleiotropic mutational effects constrain variation via purifying selection (Lande 1980; Turelli 1985; Wagner 1989; Zhang et al. 2002). These insights are especially pertinent to understanding the evolutionary maintenance of conditional phenotypes. However, said models have predominately focused on pleiotropic effects that emerge during the same organismal state (e.g. the expression of different cell types). Therefore, the model we have proposed here can be viewed as a marriage of these two perspectives, which allows for consideration of conditional phenotypes that are not constitutively expressed.

### Consistencies with and elaborations on existing theory

Predictions from analytical and simulation-based analyses provide further support and more explicit functional forms to several previous theoretical articulations. First, this model suggests the effect of pleiotropy in issuing purifying selection to conditional traits is comparable for traits that are conditionally expressed through space (Fig. 3) and time (Fig. 4). This is complementary to prior studies that suggest conditional expression through space and time has comparable effects in reducing the efficacy of purifying selection (Snell-Rood et al. 2010; Van Dyken and Wade 2010). While intuitive, this finding helps clarify the conditions under which pleiotropy is expected to play a prominent versus minor role in maintaining conditional phenotypes. Specifically, pleiotropic effects are most important when expression of a conditional phenotype is rare and become less important with increasing spatial or temporal expression probability. In other words, if a conditional phenotype is frequently expressed, this model suggests pleiotropy plays a less essential role in preventing its decay. It is important that this prediction is recapitulated because it represents the fundamental trade-off between specialization in the conditional context and deterioration of the conditional phenotype. For example, traits that are highly pleiotropic may experience sufficient purifying selection to prevent degradation but also experience constrained positive selection, thus limiting specialization in the conditional environment or genetic background (and vice versa) (Otto 2004; Guillaume and Otto 2012; Fraïsse et al. 2019; Keith and Mitchell-Olds 2019; Chen and Zhang 2020; Dapper and Wade 2020).

This model also predicts evolutionary dynamics associated with evolving pleiotropic architectures that have less prior articulation. First, our simulations suggest that an evolving pleiotropic architecture accelerates the decay of conditional phenotypes when not expressed (Fig. 5). This is biologically intuitive, as evolving trait and expression values would increase the rate that higher fitness could be achieved by alternative phenotypes (which is the governing factor when conditional phenotypes are not expressed). Exploring the pleiotropic changes that underlie these dynamics yielded elaborations on existing theory as well. Previous theoretical considerations have described how selection would favor reduced pleiotropy because it allows for greater specialization and consequently, greater adaptive potential via positive selection (Hansen 2003; Guillaume and Otto 2012). However, said considerations focus on pleiotropically linked traits that are constitutively expressed. The model we have described predicts that for conditionally expressed traits, the magnitude and direction of selection on pleiotropic associations depends on how far the initial trait values is from the alternative phenotype optimum. Specifically, when the initial trait value is less than the alternative optimum, decreased investment in said trait by the alternative phenotype (increasing *r*) is favored (Fig. 6). This is intuitive, as alternative phenotypes that decreases investment in traits that are accruing deleterious variation due to weakened purifying selection can achieve higher fitness (Fig. 6a). It is important to note that this decreased investment is afforded by lower optimal trait values relative to the initial trait value. However, if alternative optimal trait values are higher (much greater than the initial trait value), increased investment in said traits by alternative phenotypes (decreasing *r*) is favored (Fig. 6b). This represents the trade-off between expressing a trait enough to reach a high optimal value and over-investing in traits that have accrued deleterious variation due to their conditional expression pattern. When the optimal trait value is sufficiently high, the need for high expression predominates, and trait expression become more biased towards the alternative phenotype. Finally, when initial trait values are near the optimum, there exists an equilibrium point in which pleiotropic associations are sustained by purifying selection (Fig. 6b).

To our knowledge, the previously described predictions regarding the evolutionary dynamics and consequences of pleiotropic architectures are not well described or empirically evaluated. Therefore, future empirical evaluation would be useful for discerning their relevance. Furthermore, to more clearly illustrate the role of pleiotropy in the evolutionary maintenance of conditional phenotypes (the primary interest of this study), analyses were based on assumptions of high mutation rates and relatively strong purifying selection. Therefore, future exploration focusing on different evolutionary contexts (e.g. weaker purifying selection, fluctuating environments, etc.) may be useful for exploring the potential for evolved reductions in pleiotropy during periods of inexpression to fuel adaptation to a new environment, which has previously been hypothesized (Moczek et al. 2011). Finally, more recent theoretical advances have been made in understanding how discreteness of conditional phenotypes evolves (Sakamoto and Innan 2024). Therefore, future exploration of these dynamics in the model we have described here may be helpful for generalizing theoretical frameworks.

## Supplementary Material

jkaf262_Supplementary_Data

## Data Availability

All code written for running the model, output analysis, and visualization, as well as files containing the simulated data, are available on GitHub at https://github.com/gabe-dubose/geomcp/tree/main/evaluations. We also wrote a small Python package for running model simulations and investigating analytically derived functions, which is available at https://github.com/gabe-dubose/geomcp. All code and simulated data is also archived via Zenodo at https://doi.org/10.5281/zenodo.15608125. Supplemental material available at G3 online.
