# Supplementary material for: Pleiotropy and the evolutionary stability of plastic phenotypes: a geometric framework
Source: G3 (Bethesda). 2025 Nov 11;16(1):jkaf262. doi: 10.1093/g3journal/jkaf262 (PMC12774609; doi:10.1093/g3journal/jkaf262)
Supplement: jkaf262_Supplementary_Data [file jkaf262_supplementary_data.pdf]

Supplement for *”Pleiotropy and the evolutionary stability of plastic phenotypes: a geometric framework”*

Charles Qiujie Wang[a], James G. DuBose[a,*]

[a]*Department of Biology, Emory University, 1510 Clifton Rd NE #2006, Atlanta, GA 30322, USA*

[*]Issue correspondence to james.g.dubose@gmail.com

October 29, 2025

1 Derivations

1.1 Fitness function

Let X^C and X^A be binary random variables that determine whether or not an individual expresses the corresponding phenotype, such that

$$\text{Pr}(X^C = 1) = f_C, \text{Pr}(X^C = 0) = 1 - f^C \tag{1}$$

$$\text{Pr}(X^A = 1) = f_A, \text{Pr}(X^A = 0) = 1 - f^A \tag{2}$$

Letting W_0^C and W_0^A be the fitness when not expression the conditional or alternative phenotype, respectively, the realized fitness for an individual is then

$$W(X^A, X^C) = X^C W^C + (1 - X^C)W_0^C + X^A W^A + (1 - X^A)W_0^A \tag{3}$$

Therefore, the expected fitness over the stochastic phenotype is

$$\begin{aligned} \mathbb{E}[W] &= \mathbb{E}[X^C]W^C + \mathbb{E}[1 - X^C]W_0^C + \mathbb{E}[X^A]W^A + \mathbb{E}[1 - X^A]W_0^A && (4) \\ &= f_C W^C + (1 - f_C)W_0^C + f_A W^A + (1 - f_A)W_0^A. && (5) \end{aligned}$$

Since W_0^A is not applicable to our study (given $f_A = 1$) and W_0^C is 0 or constant, then relative fitness is proportional to $f_C W^C + f_A W^A$. By linearity of expectation ($\mathbb{E}[aX+bY] = a\mathbb{E}[X]+b\mathbb{E}[Y]$), $\mathbb{E}[\text{n offspring}] =$ expected fitness, which is exact for additive fitness. Therefore, the expected total fitness is

$$W^T = f_C W^C + f^A W^A \tag{6}$$

1.2 Generalized selection coefficient (S)

To derive a closed form expression of $\lambda_c(f_c, r)$, we first approximate total fitness W^T as a single Gaussian weight $W^T = \exp[-Sx^2]$, where S is the strength of selection. Recall that $W^T =$

$f_C W^C + f^A W^A$. Assuming $|x|$ is small enough that $\sigma_s\, r^2 x^2 \ll 1$ and $\sigma_s\,(1-r)^2 x^2 \ll 1$, so that all Taylor expansions below are controlled to $O(x^4)$, if $x = z - z^o$, total fitness can be defined as

$$W^T \approx f_C(1 - \sigma_s r^2 x^2) + f_A(1 - \sigma_s(1-r)^2 x^2) \tag{7}$$
$$= f_C + f_A - \sigma_s x^2[f_C r^2 + f_A(1-r)^2] \tag{8}$$

Letting $A = f_C + f_A$ and $B = \sigma_s[f_C r^2 + f_A(1-r)^2]$,

$$W^T \approx A - Bx^2 = A(1 - \frac{B}{A}x^2) \tag{9}$$

Since our goal is to approximate $W^T = \exp[-Sx^2] \implies \log W^T = -Sx^2$,

$$\log W^T = \log[A(1 - \frac{B}{A}x^2)] = \log A + \log(1 - \frac{B}{A}x^2) \tag{10}$$

For small ϵ, the Taylor expansion is $\log(1-\epsilon) \approx -\epsilon$. For our purposes, $\epsilon = \frac{B}{A}x^2$. Therefore,

$$\log(1 - \frac{B}{A}x^2) \approx -\frac{B}{A}x^2 \tag{11}$$

Therefore

$$\log W^T \approx \log A - \frac{B}{A}x^2 \tag{12}$$

Since the constant $logA$ does not effect the curvature, we take $\log W^T \approx -Sx^2$, where $S = \frac{B}{A}$. Therefore, substituting back in our terms of A and B gives the general selection coefficient

$$S = \frac{\sigma_s[f_C r^2 + f_A(1-r)^2]}{f_C + f_A} \tag{13}$$

Our primary objective is to define the evolutionary stability of conditional phenotypes that is conferred by pleiotropic links to a constitutively expressed alternative phenotype. Therefore, we can simplify this expression by assuming $f_A = 1$, expressing Equation 13 in terms of only f_C as

$$S \;=\; \sigma_s\, \frac{f_C\, r^2 + (1-r)^2}{1 + f_C} \tag{14}$$

1.3 Alternative phenotype fitness landscape integral functions

To begin exploring the consequences of an evolving pleiotropic architecture on the fitness associated with conditional phenotypes, we first conducted evolutionary simulations as previously described. However, in addition to simulations where $\sigma_r = 0$ (non-evolving r), we also conducted simulations where $\sigma_r = 0.01$. Otherwise, all parameters were set as previously described.

When conditional phenotypes are not expressed, evolutionary dynamics (in both z and r) are governed by the fitness landscape of alternative phenotypes. Therefore, to begin understanding how pleiotropic architectures are expected to evolve during periods of conditional phenotype inexpression, we analyzed how different alternative optima shape the fitness landscapes across the z and r parameter space. First, we assume a uniform trait density on a fixed window $z \in [z_{\min}, z_{\max}]$.

For the purposes of our analyses, $[z_{\min}, z_{\max}] = [-3, 3]$. The fitness landscape for alternative phenotypes for a given r is

$$I(r) = \int_{z_{\min}}^{z_{\max}} W^A(r, z)\,\mathrm{d}z \tag{15}$$

Differentiating Equation 15 for $I(r)$ with respect to r yields the marginal selection gradient:

$$\frac{\mathrm{d}I}{\mathrm{d}r}(r) = \frac{\sqrt{\pi}}{2sa^2}\big[\mathrm{erf}(u_{\max}) - \mathrm{erf}(u_{\min})\big] + \frac{-z_{\max}\,\exp(-u_{\max}^2) + z_{\min}\,\exp(-u_{\min}^2)}{a}, \tag{16}$$

Since $I(r) = \frac{\sqrt{\pi}}{2sa}\big[\mathrm{erf}(u_{\max}) - \mathrm{erf}(u_{\min})\big]$, the first term equals $I(r)/a$, so

$$\frac{\mathrm{d}I}{\mathrm{d}r}(r) = \frac{1}{a}\Big[I(r) + z_{\min}\,\exp\big(-u_{\min}^2\big) - z_{\max}\,\exp\big(-u_{\max}^2\big)\Big]. \tag{17}$$

for the boundary limit $r \to 1$ exists, we have

$$I'(1) = \lim_{r\to 1}\frac{dI}{dr}(r) = 2\,\sigma_s\,o^A\,e^{-\sigma_s(o^A)^2}\,(z_{\min}^2 - z_{\max}^2). \tag{18}$$

thus under a symmetrical window, one obtains $I'(1) = 0$.

2 Supporting results

2.1 Simulations and numerical analyses of single trait decay rates over periods of conditional inexpression

To confirm the relative importance of pleiotropic effect (r) and expression frequency (f_C) in maintaining the fitness of conditional phenotypes suggested by our deterministic model, we leveraged the exponential nature of fitness decay to numerically estimate the decay rate of conditional phenotype fitness. Specifically, the fitness of a conditional phenotype (W^C) exponentially decays with respect to the Euclidean distance from the optimum. As mutations accumulate, the distance between P^C and o^C is expected to increase linearly on average. Therefore, the expected decay in fitness associated with a conditional phenotype can be expressed as:

$$\mathbb{E}[W_t^C] \approx (W_0^C) \cdot \exp^{-\lambda_C t} \tag{19}$$

where λ_C is the decay rate. To parallel the predictions from our deterministic model, we focused on evaluating the fitness decay of single conditional traits. This allowed for tractable simulation of the evolutionary trajectories across a range of r and f_C values, which could then be used estimate the decay in conditional fitness λ_C by fitting Equation (19) to the resulting dynamics. To reduce the error associated with stochastic mutation and probabilistic sampling, we ran each simulation 10 times and fit Equation (19) to the average dynamics. Furthermore, we used Gaussian smoothing to generate a smoother contour surface of changes in λ^C across this parameter space. We performed all simulations using Python (available as supplemental information), and we used the *SciPy* Python library for all model fitting and Gaussian smoothing (Virtanen et al. 2020). As seen in Figure S1, these simulations and numerical analyses showed consistent results with that of our deterministic model.

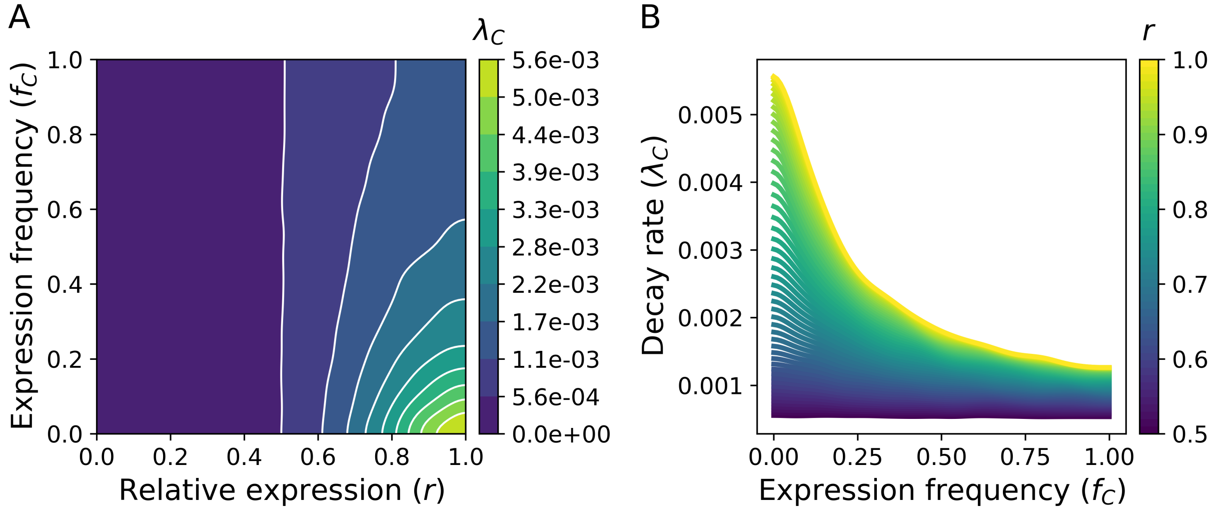

Figure S1: Numerical estimates of the role of pleiotropic constraint in shaping the fitness decay of conditional traits. A) A contour plot showing numerically estimated variation in decay rate (λ_C) as a function of relative expression in the conditional phenotype (r) and expression frequency (f_C). Lighter colors indicate greater decay rates, which occur when expression frequency is lower and relative expression is higher. B) Decay rate in conditional phenotype fitness as a function of expression frequency for traits with varying degrees of pleiotropy (r), as estimated from the λ_C surface depicted in A. Lighter colors represent higher values of r and therefore, less pleiotropy.

We used a similar approach to confirm the predictions of our deterministic model regarding how pleiotropy mediates the effect of temporal variation in expression frequency on conditional phenotype fitness decay. Here, we defined expression regimes that varied in the number of generations between expression of the conditional phenotype (g):

$$f_C(i; g) = \begin{cases} 1, & \text{if } i \bmod g = 0 \\ 0, & \text{otherwise} \end{cases} \tag{20}$$

where i is the generation index and $f_C = 1$ every g generations. We then simulated evolutionary dynamics across the r and g parameter space and estimated conditional fitness decay (λ_C) as previously described. As seen in Figure S2, these simulations and numerical analyses were again consistent with our deterministic model.

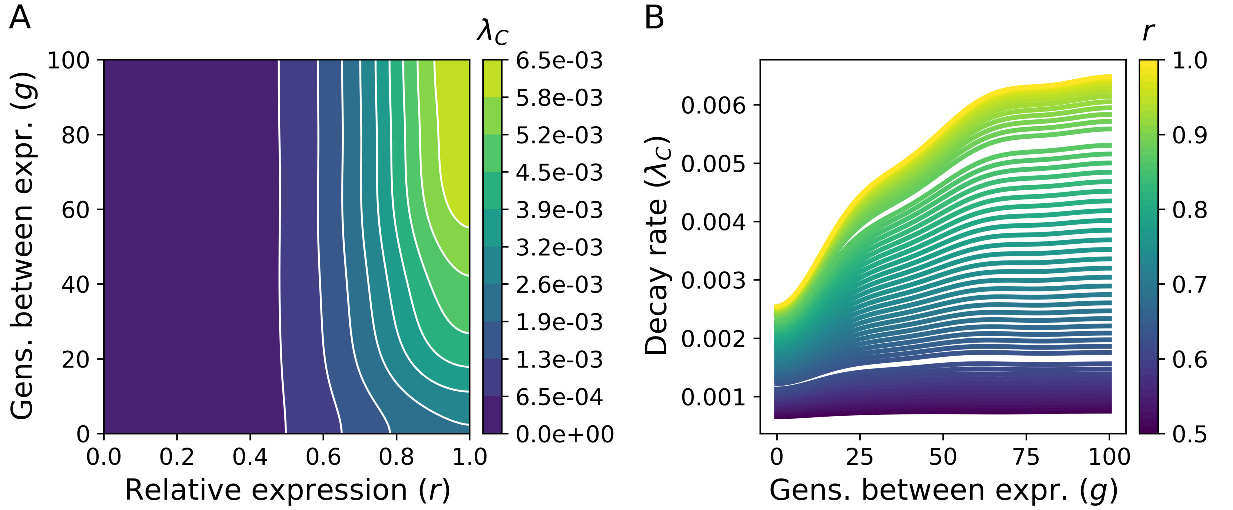

Figure S2: Numerical estimates of the role of pleiotropic constraint in shaping the fitness decay of traits that are temporally conditional. A) A contour plot showing variation in decay rate (λ_C) as a function of relative expression in the conditional phenotype (r) and generations between expression (g). Lighter colors indicate greater decay rates, which occur when relative expression is higher and there are more generations between expression. B) Decay rate as a function of generations between expression for traits with varying degrees of pleiotropy (r), as estimated from the λ_C surface depicted in A. Lighter colors represent higher values of r and therefore, less pleiotropy.

References

Virtanen, Pauli et al. (2020). *SciPy 1.0: Fundamental Algorithms for Scientific Computing in Python*. DOI: 10.1038/s41592-019-0686-2.
